# ROC study and SUV threshold using quantitative multi-modal SPECT for bone imaging

**DOI:** 10.1186/s41824-019-0057-3

**Published:** 2019-06-28

**Authors:** A. H. Vija, P. A. Bartenstein, J. W. Froelich, T. Kuwert, H. Macapinlac, C. P. Daignault, N. Gowda, O. Hadjiev, J. Hephzibah, P. Huang, H. Ilhan, A. Jessop, M. Cachovan, J. Ma, X. Ding, D. Spence, G. Platsch, Z. Szabo

**Affiliations:** 1 Molecular Imaging, Siemens Medical Solutions USA, Inc, Hoffman Estates, IL USA; 2 Friedrich Alexander Universität Erlangen, Erlangen, Germany; 3 University of Minnesota, Minneapolis, MN USA; 4 Ludwig-Maximilians Universität, München, Munich, Germany; 5 MD Anderson Cancer Center, Houston, TX USA; 6 Johns Hopkins University, Baltimore, MD USA; 7 Christian Medical College, Vellore, India; 8 Veterans Medical Center, Minneapolis, MN USA; 9 Consulting Radiology, Edina, MN USA; 10 Milwaukee Radiologists, Greenfield, WI USA; 11 Vanderbilt University Medical Center, Nashville, TN USA; 12 Siemens Healthineers GmbH, Erlangen, Germany

**Keywords:** SPECT/CT, Quantitative SPECT, xSPECT quant, xSPECT bone, Bone imaging, Concordance, ROC, AUC, SUV, Tc99m, MDP, DPD, Diphosphonate

## Abstract

**Background:**

We investigated the clinical performance of a quantitative multi-modal SPECT/CT reconstruction platform for yielding radioactivity concentrations of bone imaging with ^99m^Tc-methylene diphosphonate (MDP) or ^99m^Tc-dicarboxypropane diphosphonate (DPD). The novel reconstruction incorporates CT-derived tissue information while preserving the delineation of tissue boundaries. We assessed image-based reader concordance and confidence, and determined lesion classification and SUV thresholds from ROC analysis.

**Methods:**

Seventy-two cancer patients were scanned at three US and two German clinical sites, each contributing two experienced board-certified nuclear medicine physicians as readers. We compared four variants of the reconstructed data resulting from the Flash3D (F3D) and the xSPECT Bone™ (xB) iterative reconstruction methods and presented images to the readers with and without a fused CT, resulting in four combinations. We used an all-or-none approach for inclusion, compiling results only when a reader completed all reads in a subset. After the final read, we conducted a “surrogate truth” reading, presenting all data to each reader. For any remaining discordant lesions, we conducted a consensus read. We next undertook ROC analysis to determine SUV thresholds for differentiating benign and lesional uptake.

**Results:**

On a five-point rating scale of image quality, xB was deemed better by almost two points in resolution and one point better in overall acceptance compared to F3D. The absolute agreement of the rendered decision between the nine readers was significantly higher with CT information either inside the reconstruction (xB, xBCT) or simply through image fusion (F3DCT): 0.70 (xBCT), 0.67 (F3DCT), 0.64 (xB), and 0.46 (F3D). The confidence level to characterize the lesion was significantly higher (3.03x w/o CT, 1.32x w/CT) for xB than for F3D. There was high correlation between xB and F3D scores for lesion detection and classification, but lesion detection confidence was 41% higher w/o CT, and 21% higher w/CT for xB compared to F3D. Without CT, xB had 6.6% higher sensitivity, 7.1% higher specificity, and 6.9% greater AUC compared to F3D, and similarly with CT-fusion. The overall SUV-criterion (SUV_c_) of xB (12) exceeded that for xSPECT Quant™ (xQ; 9), an approach not using the tissue delineation of xB. SUV critical numbers depended on lesion volume and location. For non-joint lesions > 6 ml, the AUC for xQ and xB was 94%, with SUV_c_ > 9.28 (xQ) or > 9.68 (xB); for non-joint lesions ≤ 6 ml, AUCs were 81% (xQ) and 88% (xB), and SUV_c_ > 8.2 (xQ) or > 9.1 (xB). For joint lesions, the AUC was 80% (xQ) and 83% (xB), with SUV_c_ > 8.61 (xQ) or > 13.4 (xB).

**Conclusion:**

The incorporation of high-resolution CT-based tissue delineation in SPECT reconstruction (xSPECT Bone) provides better resolution and detects smaller lesions (6 ml), and the CT component facilitates lesion characterization. Our approach increases confidence, concordance, and accuracy for readers with a wide range of experience. The xB method retained high reading accuracy, despite the unfamiliar image presentation, having greatest impact for smaller lesions, and better localization of foci relative to bone anatomy. The quantitative assessment yielded an SUV-threshold for sensitively distinguishing benign and malignant lesions. Ongoing efforts shall establish clinically usable protocols and SUV thresholds for decision-making based on quantitative SPECT.

## Introduction

Bone imaging with ^99m^Tc 2,3-dicarboxypropane-1,1-diphosphonate (DPD) or methylene diphosphonate (MDP) is one of the main pillars of nuclear medicine for oncology and orthopedic applications (Buell et al. 1982; Lantto et al. 1987). Single-photon emission computed tomography (SPECT) with these tracers has superior clinical performance to two-dimensional (2D) scintigraphy (Even-Sapir et al. 2009). Results of a recent review article suggest that the advent of integrated, in-line SPECT/CT with diagnostics-capable computer tomography (CT) systems further improves SPECT performance relative to scintigraphy (Kuwert 2014). However, the detection and precise localization of small lesions remains challenging due to the comparatively low SPECT resolution, which is limited to about 8–12 mm by the collimated image formation. Furthermore, previous SPECT methods suffer from a lack of standardized absolute quantification, although they suffice to obtain absolute quantitative uptake at a given clinical site (Zeintl et al. 2010). However pooling data between sites is very challenging or indeed impossible when uncertainties propagate cumulatively from multiple sites without standardization to a global reference. We intended to overcome these limitations of bone SPECT by first developing a quantitative reconstruction with a standardized calibration and then exploiting anatomical information derived from the CT modality as already installed in contemporary general purpose SPECT/CT systems.

From the perspective of nuclear medicine imaging, the anatomical information derived from CT data is designated “extra modal information” (EMI) to distinguish it from the “intra-modal information” (IMI) extracted from SPECT data. As early as the 1990s, proof-of-concept research showed enhancement of SPECT images by incorporating anatomical information into the SPECT reconstruction, e.g., (Calvini et al. 2001; Gindi et al. 1993). These early efforts suffered from the potential pitfall that the anatomical information could create clinically relevant artifacts in the emission image, or otherwise interfere with the reader’s clinical performance, a limitation further compounded by their considerable computational expense using the technology of the day.

Current clinical practice is to show SPECT/CT multimodal images in a fusion display. Between 2008 and 2013, Siemens Medical Solutions USA, Inc., Molecular Imaging (SMS MI) developed a new reconstruction method that incorporates anatomical information from a CT scan into the SPECT reconstruction and circumvents earlier pitfalls, while also yielding absolute quantification relative to a reference standard. At Siemens SPECT Research, our working definition of absolute SPECT quantification is the ability to measure activity concentration distribution in three-dimensional (3D) volumes, along with the corresponding uncertainty distributions. Here, the most fundamental aspect of quantitation is enabled by linking to a reference standard for both the imaging device and the dose calibrator.^1^ A sharp delineation between anatomically distinct tissue types is obtained by automatic segmentation of the CT-derived high-resolution adaptive linear attenuation map (Vija et al. 2005). This approach yields zone maps, wherein the criteria for defining particular zones need to be justified for specific applications. In general, a zone is comprised of voxels with the same or similar CT signal attenuation. The zone images have a very striking and distinctive appearance, amalgamating the sharp tissue delineation inherent to CT with the soft uptake gradients and noise structure typical of emission data. This hybrid imaging method is ultimately based on the protean properties of any specific tissue class, with detection obtained by a range of imaging modalities. In the present context, the reconstruction algorithm is designed to mitigate against unjustified zonal segmentation, as the reconstruction method is driven by the emission data only.

Bone SPECT imaging with ^99m^Tc-DPD/MDP was chosen as our first application of this methodology. This choice is appropriate, as diphosphonate tracers bind to bone but not soft tissues, thus specifically depicting bone metabolism and turnover. Bone SPECT clearly requires segmentation between bone and non-bone voxels, which are readily distinguished by CT due to the high contrast in tissue attenuation. xSPECT Bone is a processing procedure designed specifically for diphosphonate imaging, wherein the CT-based segmentation of bone and non-bone tissue classes fulfills a necessary condition for the method (Vija and Yahil 2015; Vija 2013; Vija 2016; Vija 2017). Furthermore, motion and registration issues are less pronounced for bone, which is amenable for rigid registration methods. The image reconstruction is designed to work in the CT-defined reference frame and developed algorithmic steps to minimize further potential pitfalls in registration.

In this test of our new reconstruction concept, we undertook a multi-center clinical study with indications for bone metastases of either breast or prostate cancer. Our analysis entails both visual and quantitative assessments. We describe global image quality and concordance between readers in lesion characterization, and establish a surrogate truth to determine the receiver operating characteristic (ROC) curve for classification. Finally, we employ area under the curve (AUC) analysis and compute a standard uptake value (SUV) threshold based on absolute lesion uptake. The prototype software used in this work has since been developed into commercially available Siemens products: xSPECT calibration source kit, xSPECT Quant™, xSPECT Bone™, and the Symbia Intevo SPECT/CT product line. The commercialization process ensures the equivalence to the clinically tested prototypes reported herein.

## Methods

### Patient inclusion and exclusion criteria

We assembled data from patients who had undergone bone imaging for metastatic prostate or breast carcinoma. Each patient received a nominal activity of 740 MBq ^99m^Tc-MDP or -DPD, depending on the site’s clinical practice, and had given informed consent for their participation in the research studies, which were approved by local investigational review boards (IRB). The patients underwent preparation according to the routine clinical protocol at each site. Data was acquired at three sites in the USA and two sites in Germany over a period of 30 months. For the first 12 months, we randomly selected data for algorithm development, pilot evaluations, and study design tests; these pilot subjects were excluded from the main study, as were any data used for research projects other than the present study. We also excluded data exhibiting visible motion in the projections, or with CT artifacts due to metal implants or other CT-related technical issues. We qualified 76 SPECT/CT data from 72 patients (37 male), of whom four had two-bed scans, and had undergone thorax and/or abdomen clinical SPECT examination. 65/72 (90%) of the scans were acquired in the interval 12–24 months after the start of data collection, with the remaining seven cases being from the first six months, in accordance with the requirements of an initial power analysis. All five sites used standard dose or weight-based injected dose adaptation, according to their clinical practice. The targeted post-administration imaging time was 3 h. Since there is no known clinical difference between the two radiopharmaceuticals, we pooled the data (Lantto et al. 1987).

Table 1 shows the patient and data descriptive statistics; patient weights ranged from 53 to 127 kg, and the injected dose from 350 to 1006 MBq either of MDP (51 patients in US) or DPD (25 patients in Germany). The total counts ranged from 4.8 to 11 Mc (million counts) for thorax scans and 2.5 to 11 Mc for abdomen scans.

**Table 1:** Summary of patient demographics and SPECT/CT acquisition parameters

Statistics	Mean	SD	Median	Minimum	Maximum
Female (39 data; 35 patients)					
Height (cm)	162.10	11.6	164.0	110	175
Weight (kg)	72.1	14.5	70.0	52	127
BMI	27.0	5.2	26.2	19	42
Injected dose (MBq)	744.6	168.6	728.0	336	1000
Dwell time (s)/ view	13.3	2.3	13.0	9	17
CT tube current (mA)	101.3	80.7	65.0	22	276
Male (37 data; 37 patients)					
Height (cm)	177.87	6.8	180.0	163	189
Weight (kg)	84.4	17.8	82.0	24	122
BMI	27.1	4.5	26.0	21	41
Injected dose (MBq)	791.9	136.6	777.0	481	1006
Dwell time (s)/view	13.5	2.0	14.0	9	17
CT tube current (mAs)	82.1	71.8	50.0	24	266

### Lesion selection criteria

In each SPECT volume, three lesion candidates were defined and labeled by a nuclear medicine physician otherwise uninvolved in the reading process. Planar whole body scans, conventional iterative SPECT/CT reconstructions (Flash3D), CT, and the clinical report were available for lesion definition; reconstructions using the new algorithm were not used at this stage, thus avoiding bias with respect to lesion selection. We calculated the statistical power and necessary distribution of lesion classification through pilot research in support of the greater study (see Vija 2013 for details). The intended distribution of lesion classification was 40% malignant, 40% benign, 10% questionable (with respect to existence, not to malignancy), and 10% negative controls. When possible, the three defined lesions belonged to different lesion classes and resided at different locations (e.g., for SPECT/CT, lesions were not only in vertebral bodies, but also in ribs or the pelvis). Description of location and initial classification were noted, but not reported to the readers.

### Reader selection

From each of the five institutions, we recruited one principal reader, and four institutions added at least one more reader, representing a wide range of clinical experience in use of the Siemens equipment. The principal readers were the most experienced, but all recruited additional readers were board certified in Nuclear Medicine in their respective countries and were employees of their department. One reader also had a certification in Radiology.

### Imaging and quantitative calibration

We used six SPECT/CT systems (Symbia T2, T6, or T16 with 2, 6, or 16 slice CTs, SMS MI, Hoffman Estates, Il, USA) at the five participating sites, with research software (VA63). All systems were factory serviced and maintained to ensure optimal clinical product image performance. Each site employed a newly designed ^57^Co point source to serve as a global reference standard. In a calibration step, the source was positioned at the center of the FOV of both detectors and then extended to the maximum radial position, thus illuminating both detectors equally. This same ^57^Co source was also used to cross-calibrate each site’s dose calibrator for measurement of the injected dose. The source design specifies a nominal activity of 111 MBq, accurate to within ± 3% (99% CL or 2.56 σ) National Institute of Standards and Technology (NIST)-traceable uncertainty of the known manufactured strength, which itself resides within a 15% acceptance range. The quantitative xSPECT reconstruction is designed to estimate the absolute radioactivity concentration within the reconstruction process, yielding voxelwise images in units of Bq/ml, decay-corrected to the injection time.

#### Acquisition

The participating sites had aligned their respective clinical protocols, extending from patient preparation to the acquisition protocol, at the start of the collaborative project. Thus, all data were acquired with a standard Siemens Autoform® LEHR collimator, a 15% energy window centered on 140.5 keV, and a 15% lower energy scatter window adjacent to the peak window. A whole body planar scan was acquired, as well as a SPECT scan with either one or two bed positions using 120 views over 360° in a non-circular close patient orbit, with a 256 matrix and 2.4 mm isotropic pixel size, and a default dwell time of 20 s per view. The VA63-Symbia systems are configurable to acquire both conventional framed data and list mode data, thus allowing simultaneous data acquisition for both clinical and research use, without needing a dual scan. The CT was acquired prior to the SPECT scan, using CareDOSE 4D® with the site-specific selection of the effective currents ranging from the lowest possible dose setting (17 mA) to diagnostic quality CT dose (200 mA). The rotation time of the CT was set to the fastest possible, either *T* = 0.6 s (T6, T16) or *T* = 0.8 s (T2), with a pitch of unity to minimize CT motion artifacts. In addition to the site’s typical CT reconstruction kernels, we chose the diagnostic quality Siemens B31s kernel (where B is for Body kernel) as the preferred kernel for both attenuation and multimodal applications, such as xSPECT Bone. The B31s CT kernel for the T6 has a theoretical characteristic value of the modulation transfer function (MTF) M(ρ) at 50% M(ρ_50_) = 3.60 1/cm, and with *M* ≤ 1, for all ρ. This kernel yields a good compromise between high resolution and noise, and is therefore well-suited for attenuation correction and zone map generation. The CT doses vary in this trial to address a wide range of clinical practice. We believe that such CT images can be used as follow-up to establish the surrogate truth as described below. We are not aware of a systematic trial comparing low vs. high dose CT performance for prostate and breast cancer. However, we cite Spira et al. as follow-up gold standard technique, as well as the work of Gleeson et al. comparing low dose CT to planar skeletal survey in myeloma, which showed that low dose CT is better than skeletal survey (Spira et al. 2012; Gleeson et al. 2009).

#### SPECT/CT reconstruction

We used Flash3D (F3D) as the reference method for reconstruction (Vija et al. 2003; Roemer et al. 2006). We reconstructed F3D in both 128^2^ and 256^2^ matrices, with isotropic voxel sizes of 4.8 and 2.4 mm, respectively. In a pilot trial using a random sample of subject data, we had found no difference in clinical performance between 128^2^ and 256^2^ F3D images (Research Documentations n.d.). Nonetheless, for the present evaluation, we consistently used the clinical 128^2^ matrix and best clinical practice F3D reconstruction parameters for subset, iteration, and post-smoothing of each dataset. This decision ensured that the reference reconstruction resulted in the best possible image quality, and was customary at all clinical sites. The new reconstruction method is based on conjugate gradient (CG) and uses “zone maps” derived from CT, which maintain sharp tissue boundaries (Vija and Yahil 2015; Vija 2013; Vija 2016). We here designate this method as either conjugate gradient, attenuation and scatter (CGAS) or conjugate gradient, zonal, attenuation and scatter (CGZAS), where the A and S denote attenuation and scatter correction, and Z denotes the use of zones for extra modal information (EMI). The now commercially available xSPECT Quant (xQ) is based on CGAS, and xSPECT Bone (xB) is based on CGZAS. We obtain the zone map from a threshold-based segmentation of a 140 keV reference linear attenuation coefficient “μ-map,” which is derived from the CT using a B31s CT reconstruction kernel. Five tissue classes (“zones”) are segmented: cortical bone (zone #5), spongy bone (zone #4), soft tissue (zone #3), adipose tissue (zone #2), and lung/air (zone #1). All CG reconstructions were done in a 256^2^ matrix, while the CT is in a 512^2^ matrix. Thus, each SPECT Voxel contains four CT voxels. We performed the SPECT reconstruction in the anisotropic space of the CT frame-of-reference, thus ensuring optimal SPECT-to-CT image data registration. However, we used post-reconstruction isotropization of voxels prior to disposition of reconstruction results, such that the F3D and xS images are more accurately displayed in the same reference frame. The μ-map to zone map conversion is smooth, containing also non-integer values to represent tissue mixing in a SPECT voxel, which is accommodated by a nonlinear interpolation. The attenuation and scatter correction method is identical in all reconstructions (Vija et al. 2005; Vija et al. 2004; Ichihara et al. 1993; Ogawa et al. 1991). However, Siemens internal research has shown that the scatter projection estimate (SPE) must be smoothed with a 2D Gaussian kernel with 15 mm FWHM to minimize noise propagation (Research Documentations n.d.). The current clinical standard of acquiring larger pixels in a 128^2^ matrix does not typically require such noise reduction operation. The choice of reconstruction parameters was determined by a two-alternative forced choice evaluation where the readers were asked to evaluate for reading preference and general image quality data sets from eight subjects who were not included in the final concordance evaluation. We evaluated the number of updates and subsets for varying dose levels, and smoothing schemes (Ma et al. 2013), and compiled and analyzed the resulting data to determine a range of reconstruction parameters, finally arriving at common reconstruction parameter settings for all data. For total counts exceeding 6 Mc, we choose 48 updates (48 iterations, and one subset) with a FWHM of a 3D Gaussian post smoothing of 10 mm for all tissues in the case of CGAS, or 5 mm in bone zones and 10 mm in non-bone zones with zonal masking preserving the edge boundaries in zonal reconstructions. For studies with total counts less than 6 Mc, we used only 24 updates (24 iterations and one subset). We note that resolution recovery and noise build-up behavior of CG differs from that of OSEM. In a separate study, we found that four OSEM updates corresponds roughly to one CGM update (Ma and Vija 2014a; Ma and Vija 2014b), which is consistent with a previous finding (Tsui et al. 1991). All studies used automatic rigid registration based on full field-of-view (FOV) mutual information, as a second step after calibrated registration between the CT and SPECT FOVs to mitigate against potential intra-scan patient displacement.

#### Anonymization

The data acquired on each SPECT/CT system were transferred to the advanced research computer (ARC), which is only accessible to collaboration partners, thus maintaining strict separation of clinical and anonymized research data. The subsequent use of any research software is disabled unless the data header is anonymized, removing patient identifying information and renaming the dataset with a random name. Each research site created and maintained a key file, which is not accessible to Siemens researchers. The anonymized data is then transferred automatically to a separate hard drive on the ARC and integrated into two different databases, which are visible to the Syngo® data browser and research database tool. Data included in the evaluation described herein underwent a second anonymization step to remove any remaining site and reader identifiers. The reader anonymization keys are only accessible to the Siemens research team.

### Evaluation and questionnaire design

All primary readers were involved in the development of the prototype providing feedback and image quality preference on a small set of data sets, which were not part of this study. All other readers from the respective institutions had their first exposure to the images during the training phase for the study, which serving to balance out potential bias. In general, great care was taken to ensure as unbiased a test as possible. However, the multi-modal reconstruction xB results in images having an appearance clearly distinct from reconstructions not using the CT data, such as EMI. Thus, a strictly blinded study design is impossible; our goal was to ascertain the clinical performance of these new images, without bias due to the unmistakable xB images. We attempt to answer the following questions: Did readers like the new images? Did they read with less variability? Were the readers more confident? And finally, how correct were the readings as compared to “truth”? To answer these questions, the study design entailed two main parts: (a) blinded global and local assessments, and (b) unblinded read for consensus analysis to define the reference “truth” in support of an ROC analysis. In part (a), we assessed the readers’ subjective responses to the image appearance, and then their ability to detect lesions and classify them as either malignant or benign. This judgment was obtained in two steps, first from inspection of the images over the entire FOV and then by reading specific regions that may or may not have contained lesions.

#### Read sequence and display

**Fig. 1:**
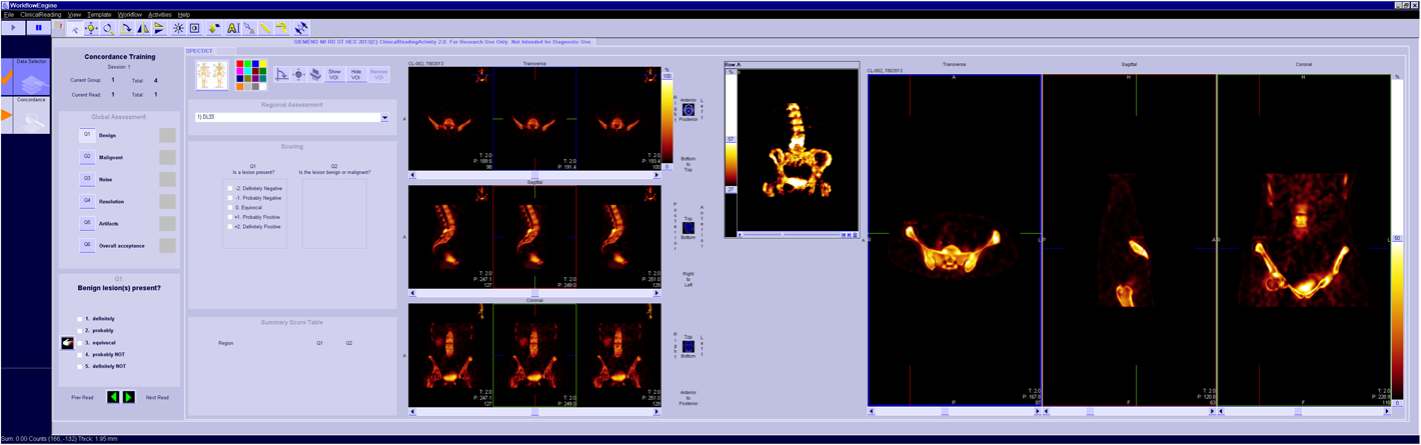
The user interface of clinical evaluation software

Each reader had to read four variants for each scan, namely (i) F3D, (ii) F3D fused with CT, (iii) xB, and (iv) xB fused with CT. There were 72 subjects in total, with 76 scans and assessment of three VOIs each, such that there was a grand total of 4 × 76 × 3 = 912 reads. Based on preliminary tests, the average read took about 4 min, thus totaling 60 h. The reads were undertaken in separate sessions to minimize the variance of read results due to fatigue, and to avoid the “Friday afternoon syndrome”, where readers hurry to complete the final reads in a block. Such effects would have biased the results, based on our observations in pilot trials used for designing this study. As a counter measure to ensure conditions as equal as possible, we split the reads into four sections of 22 reads; each section started with a training session of three reads to accustom the reader to the changing visual impressions of image quality of the subsequent variants of image reconstruction. The data sequence was randomized and reads of the same subjects were distributed as widely as possible across all reading sessions to minimize potential memorization of subject images, especially of the distinctive common CT. We also randomized across all readers, to avoid potential cross-contamination, were readers to discuss sessions or data among themselves. We developed an algorithm that defined a random read sequence for all studies and all readers, given the rules of maximal distribution, and no repeats of subjects within a session. Suspension of a read session was allowed and every data entry and read duration was recorded. We prepared a special graphical user interface (GUI) (Fig. 1), using only graphical display components already customary from the clinical setting, and affording analysis and visualization of the images. We presented images in a three-slice display from top to bottom as transverse, sagittal, and coronal views. We also provide maximum-intensity-projection (MIP) images to the right of the SPECT and CT in transverse, sagittal, and coronal views, showing a fusion image and lesion identification markers, depending on the stage of the evaluation. The readers set the color schemes to their individual preference, thus maximizing each reader’s performance. We used two side-by-side 24″ monitors calibrated using test images with resolution of (1920 × 1080) pixels, and the environmental conditions were adjusted to be the same as in clinical read conditions and to be similar at all sites.

#### Alignment of reader’s interpretation of questions and gradations

The study design relies on the reader’s interpretation of the meaning of words to assign a five-point scale in answer to a question. Reader training and agreement prior to the study is thus necessary to align the readers, since internal pilot studies showed high variance in responses that was unrelated to the response to the image itself. Therefore, all readers (both native and non-native English speakers) obtained training through a common discussion of the meaning of the words, and referring to example images. Prior to agreement about the phrasing of the questions and answers, we implemented a consistent order of the answers, namely that the first answer is always favorable to the question and the last answer unfavorable. Finally, we conducted test runs to ensure that all readers show sufficient commonality in their interpretation of the questions and similar thresholds for judging nuances between the five point gradations.

#### Visual assessment of global image quality

We first evaluated each reader’s ability to detect and classify a lesion anywhere in the reconstructed volume with the questions: (1) benign or (2) malignant “lesion(s) present?”, with possible answers as follows: (1) definitely, (2) probably, (3) equivocal, (4) probably NOT, and (5) definitely NOT. We then assessed the clinical impression as well as amenability to use the image in clinical practice, with an overall image quality assessment of the entire FOV for: (A) noise: “Please rate the general impression of the background noise”, with possible answers: minimal, low, fair, poor, severe (risk of wrong diagnosis). (B) For structural resolution we asked, “Please rate the general impression of the structural image resolution” with possible answers: excellent, very good, good, fair, poor. (C) For presence of artifacts, we asked, “How severely do artifacts impair interpretation”, with possible answers: not at all, minimal, low, moderate, severe. (D) For overall acceptance, we asked, “Overall, do you accept this SPECT image and would you use it in your clinical practice?”, with possible answers: strongly yes, probably yes, do not know, probably no, strongly no.

#### Visual assessment of lesion detection and classification

**Fig. 2:**
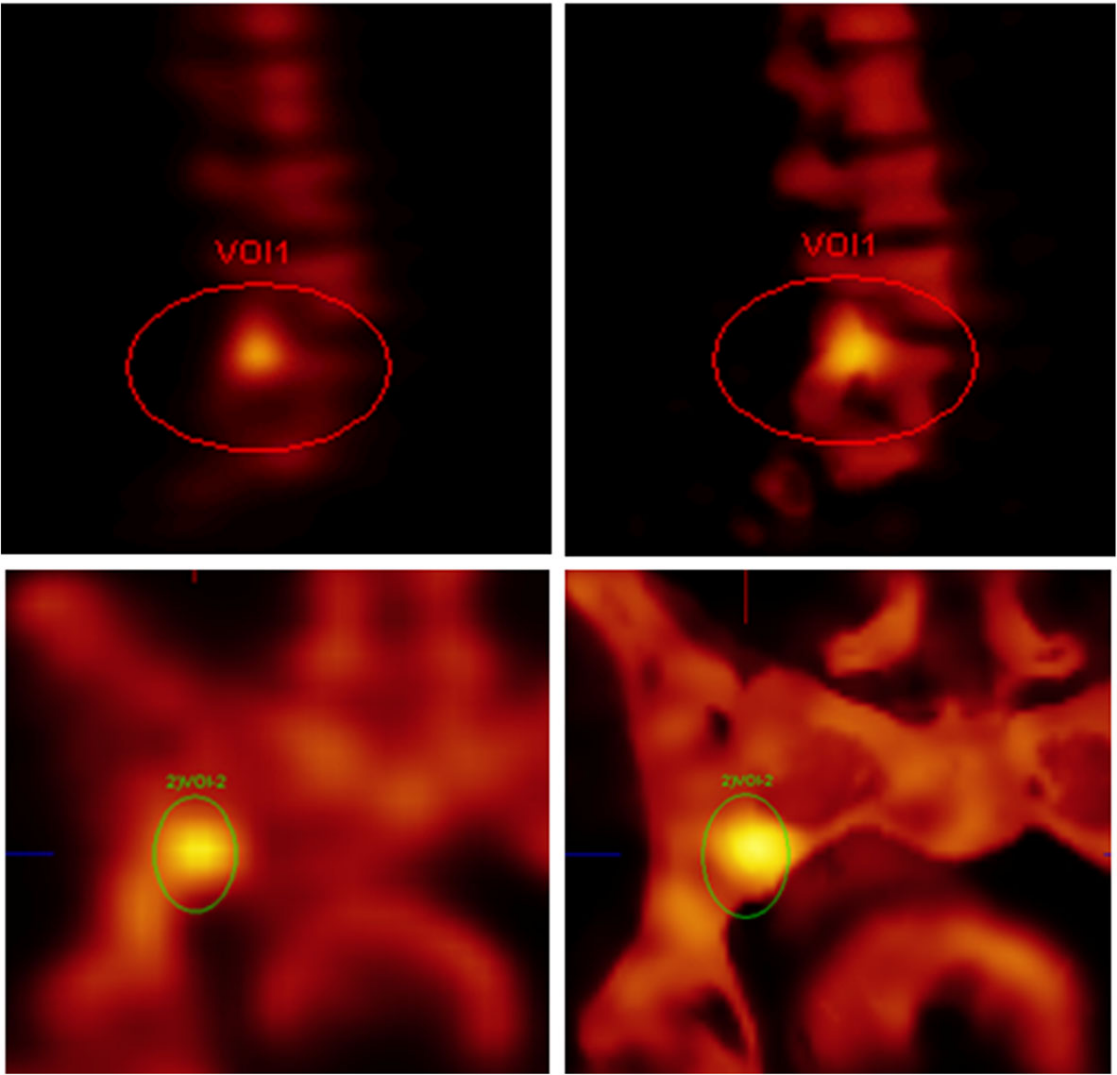
Examples of VOI placed on bone for F3D (left) and xB (right)

We then evaluated the reader’s ability to detect and characterize a lesion in a specific bone location. The location was marked on the screen by a circle, designated by anatomical name, the number of the bone, and left-right, if applicable. Such marking was intended to minimize potentially false identifications of the lesion in question, by preventing fatigue or distractions contributing to error rates. Lesion selection was performed by a board-certified Nuclear Medicine physician not otherwise involved in the reading tasks, but having access to all clinical data, including the anonymized clinical report. Three potential lesions in each of the 76 SPECT volumes were labeled as ellipsoid VOIs in the F3D reconstructed SPECT/CT fusion image (Fig. 2). To avoid any selection bias, we did not use the new multimodal SPECT reconstruction in this step. Using the available information, 228 lesions were classified as malignant (25%), benign (36%), definitive lesions of unclear malignancy (5%), borderline lesions, i.e., faint “warm” spots (8%), in need of further review (10%), or negative controls (16%). This “on-the-fly” classification was neither shown to the readers nor used for further evaluation, but only served to guarantee a reasonable distribution of lesion types. In addition, we annotated the lesions by a location parameter to separate “joint” from “non-joint” locations, thus enabling a subsequent constrained analysis. This annotation was not shown to the readers, but is very apparent in the CT fusion images. The criteria were not specified, but each reader applied established criteria and own experience. Example criteria for malignancy included random distribution, localization to bones of pelvis and lumbar spine in prostate cancer, or a nonlinear shape not following joint surfaces, and that joint-related foci are usually benign.

### Surrogate truth reading

The readers reviewed the reconstructions together with the CT image and all available information. Images from xB and F3D reconstructions generated separate truth reads, designated as “xB-truth” and “F3D-truth.” The surrogate truth model was constructed by clustering the reads into two categories according to majority vote of the now nine participating readers to determine F3D-truth and xB-truth. Discrepancies between F3D- and xB-truth were reviewed using all available information and the final truth was assigned by consensus. The blind readings of F3D, F3D/CT, xB, and xB/CT were compared against that final truth, from which we calculated sensitivity and specificity, plotted the ROC curves, and computed AUC and critical values.

#### Concordance and quantitative uptake analysis

For the analysis of global image quality, we used the mean difference of ratings to calculate the Pearson’s correlation with Yates correction, ${\upchi}_{\mathrm{Yates}}^2$. As a measure of confidence, we calculated the likelihood ratio, *R*_C_ = (*H*_xB_/*L*_xB_)/(*H*_F3D_/*L*_F3D_), where *H* counts when the lesion was read confidently positive or negative (+ 2, − 2), and *L* counts when the lesion was equivocal (− 1, 0, 1). Table 3 shows the correlation table, where the total number of lesion cases is 2052 from nine readers of 76 data sets, each containing three lesions tallied within H and L strata. Table 4 is the subsequent analysis computing the confidence likelihood ratio. We used the aforementioned VOIs to create lesion volumes based on a threshold of 50% of maximum intensity in that VOI, for which we then computed the descriptive statistics. The intensities were in units of kBq/ml, which we also converted to SUVs, knowing the injected dose and patient body weight. All variants of SUV could be computed, but we here present only the mean, body weight-based SUV. No dedicated partial-volume correction (PVC) was applied to the SUVs.

## Results

### Image quality and reader concordance assessment from visual interpretation

The results of the global visual assessment are summarized in Tables 2 and 3, whereas Table 4 presents the reader confidence results. Based on the five-point scale for image quality ratings, xB had resolution deemed almost two points higher, while the overall acceptance was one point higher as compared to F3D. The confidence level to characterize the lesion was significantly higher with xB than with F3D (3.03x w/o CT, 1.32x w/CT). In lesion detection and classification, there was a high correlation between xB and F3D scores, but lesion detection confidence increased by 41% without CT, and by 21% with CT when using xB as compared to F3D.

**Table 2:** Global image quality rating

	Mean score: F3D	Mean score: xB	Difference score (xB-F3D)	Std. error	Mean score: F3D/CT	Mean score: xB/Ct	Difference score (xB/CT-F3D/CT)	Std. error	Conclusion
Background noise	2.22	1.94	− 0.29	0.045	2.04	1.80	− 0.25	0.046	xB better, *p* < 0.001
Structure resolution	3.49	1.72	− 1.77	0.049	3.18	1.50	− 1.68	0.052	xB better, *p* < 0.001
Artifacts severity	2.47	2.11	− 0.36	0.057	2.11	1.88	− 0.23	0.053	xB better, *p* < 0.001
Overall acceptance	2.63	1.73	− 0.91	0.054	2.41	1.54	− 0.87	0.053	xB better, *p* < 0.001

Score: the lower score number the better

**Table 3:** Lesion-based correlation and confidence likelihood ratio analysis I

Q1: Lesion detection							Q2: Lesion classification					
	Without CT			With CT			Without CT			With CT		
	xB high	xB low	Total	xB high	xB low	Total	xB high	xB low	Total	xB high	xB low	Total
F3D high	1103	141	1244	1290	135	1425	156	79	235	706	157	863
F3D low	303	505	808	215	412	627	392	963	1355	270	509	779
Total	1406	646	2052	1505	547	2052	548	1042	1590	976	666	1642

**Table 4:** Lesion-based correlation and confidence likelihood ratio analysis

	Q1 Lesion detection			Q2 Lesion classification		
	Value	*p* value	Conclusion	Value	*p* value	Conclusion
Correlation: χ_Yates_^2^						
w/o CT	595	*p* < 0.001	Correlation high	124	*p* < 0.001	Correlation high
w/ CT	704	*p* < 0.001	Correlation high	378	*p* < 0.001	Correlation high
Confidence likelihood ratio *R*_C_						
w/o CT	1.41x	*p* < 0.001	Confidence increased 41%	3.03x	*p* < 0.001	Confidence increased three folds
w/ CT	1.21x	*p* < 0.01	Confidence increased 21%	1.32x	*p* < 0.001	Confidence increased 32%

Note: χ_Yates_^2^ (0.001, 1) = 10.83, calculation of *R*_C_ example (548/1042) / (235/1355) = 3.03x

We first had to establish that the construction of the surrogate truth was sound. A cross-check of the null hypothesis that the surrogate truths based on a F3D or xB readings are identical cannot be rejected, yet the unblinded truth reads clearly outperformed the reads without all available information (*p* < 0.05). This indicates that our unblinded construction of surrogate truth using all available information of the consensus reads from all readers is plausible.

Table 5 shows the summary of specificity and sensitivity, indicating an improved overall reading accuracy for methods using EMI compared to the standard method. We assess reader agreement by the intraclass correlation coefficient (ICC) (Fleiss and Shrout 1977). Table 6 summarizes the ICC and 95% confidence level for absolute agreement between the readers. Table 7 shows a correlogram between the decisions using the methods and the final decision. These tables show good agreement between readers and the decisions they made, with the least agreement occurring when reading only the F3D. Reading only xB yielded similar results when also showing images fused with CT.

**Table 5:** Sensitivity and specificity

Modality	Sensitivity (%)	95% CI	Specificity (%)	95% CI
F3D	68.29	64.8–71.6	88.96	87.1–90.6
xB	72.76	69.4–75.9	95.28	94.0–96.4
F3D/CT	81.71	78.7–84.4	93.91	92.5–95.1
xB/CT	82.38	79.4–85.1	95.66	94.4–96.7
Comparison of area under the curve (AUC)				
Modality	AUC	SE^a^	95% CI^b^	Sample size
F3D	0.786	0.0114	0.768 to 0.804	2052
F3D/CT	0.878	0.0092	0.863 to 0.892	Positive group
F3D “Truth”	0.994	0.00234	0.989 to 0.997	738
xB	0.84	0.0105	0.824 to 0.856	Negative group
xB/CT	0.89	0.0089	0.876 to 0.903	1314
xB “Truth”	0.988	0.00329	0.982 to 0.992	

^a^Hanley and McNeil (1982, 1983)

^b^Binomial exact

**Table 6:** ICC and 95% confidence level for absolute agreement

Absolute agreement		Intraclass correlation^a^			
		F3D	F3DCT	xB	xBCT
Intraclass correlation^a^	Single measures^b^	0.48	0.67	0.64	0.7
	Average measures^c^	0.89	0.95	0.94	0.95
95% Confidence interval	Single measures^b^	0.43 to 0.54	0.63 to 0.72	0.60 to 0.69	0.65 to 0.74
	Average measures^c^	0.87 to 0.91	0.94 to 0.96	0.93 to 0.95	0.94 to 0.96

Shrout PE, Fleiss JL, 1979

^a^The degree of consistency among measurements

^b^Estimates the reliability of single ratings

^c^Estimates the reliability of averages of 9 ratings

**Table 7:**
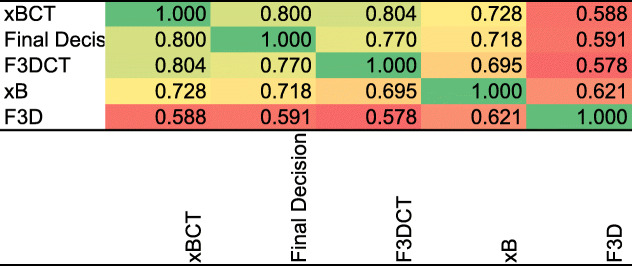
Correlogram between the decisions

The pairwise comparison of the ROC curves shows that all curves differ significantly, except for the case when SPECT-CT fusion images are read (*p* < 0.05). This is unsurprising, since the truth reading post evaluation and the initial truth in the definition stage used the F3D SPECT image and CT. When reading SPECT scans without CT, xB had 6.6% higher sensitivity, 7.1% higher specificity, and 6.9% greater AUC compared to F3D, indicating that xB indeed improves diagnostic accuracy. This accuracy is further validated against the current gold standard (F3D/CT) when xB is fused with CT.

### Image examples

**Fig. 3:**
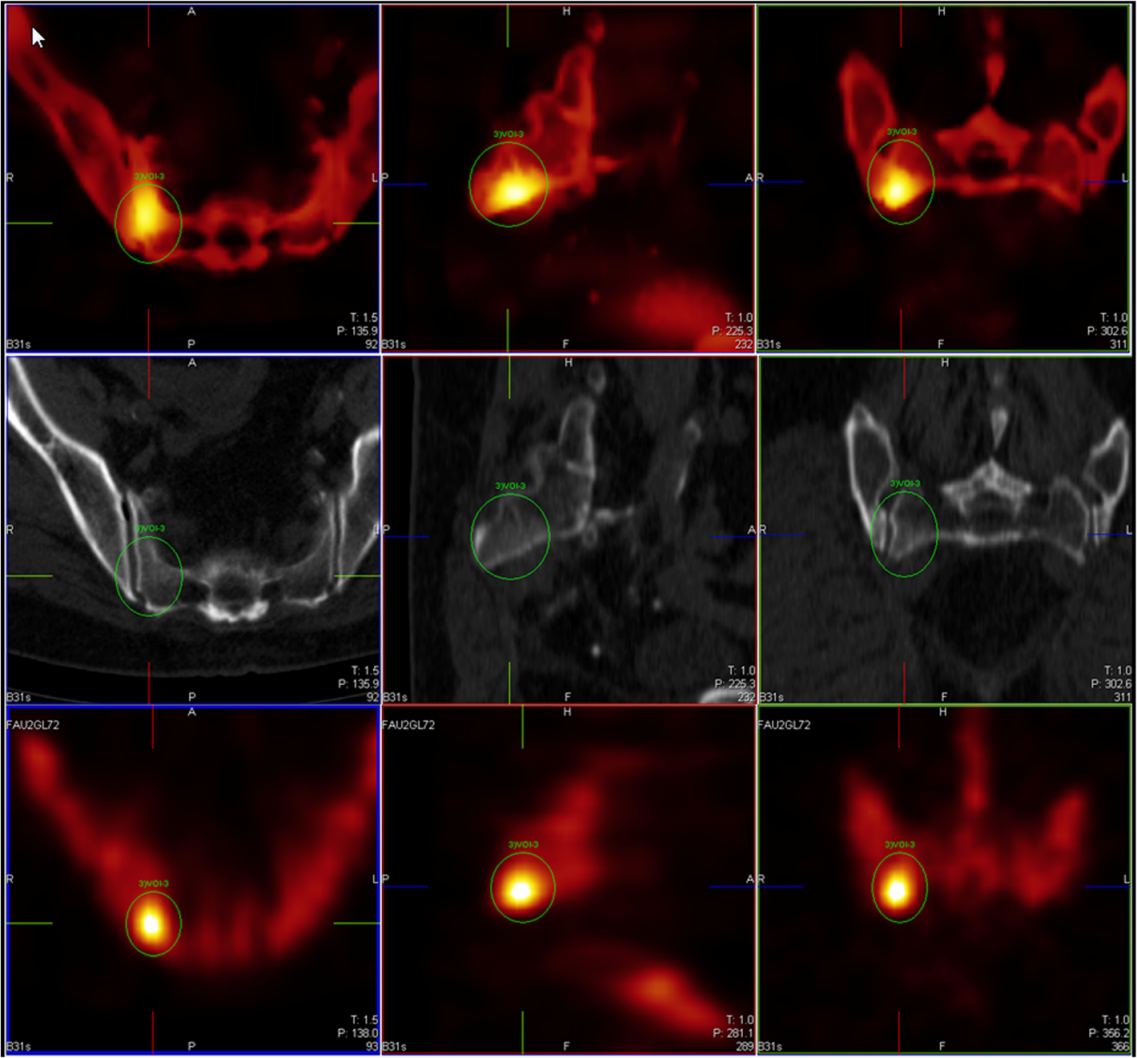
Reduction of false negative reads with use of xB. In this case, two of nine F3D reads were deemed benign and thus false negative, while all nine xB reads were as malignant, in agreement with the surrogate ground truth read. [FAUGL72 L3]

**Fig. 4:**
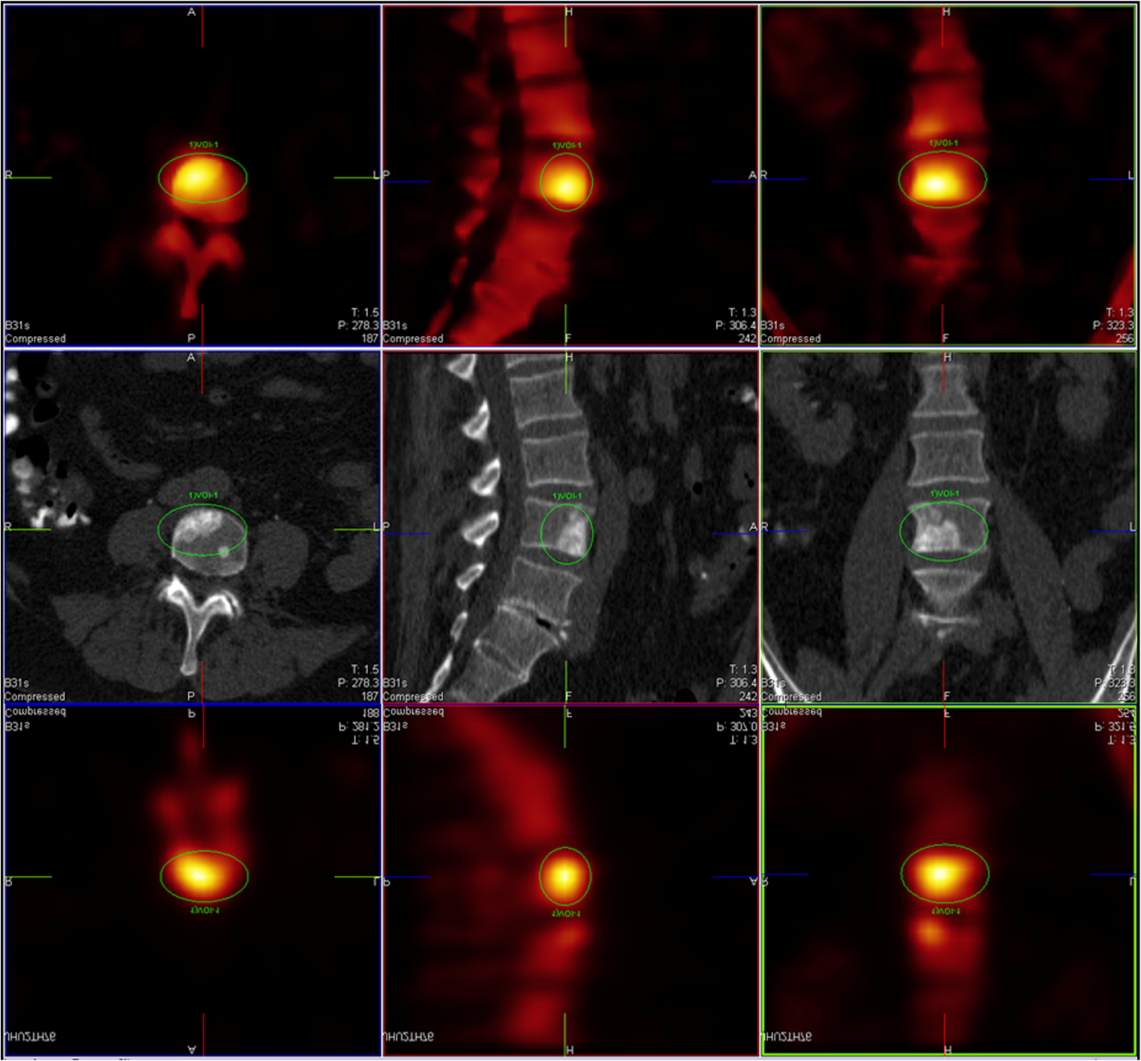
Reduction of false negative reads with xB. Here, a clearly visible lesion, read as benign by two readers in F3D, and as malign by all readers in xB, with confirmation by CT. [JHUTH76 L1]

**Fig. 5:**
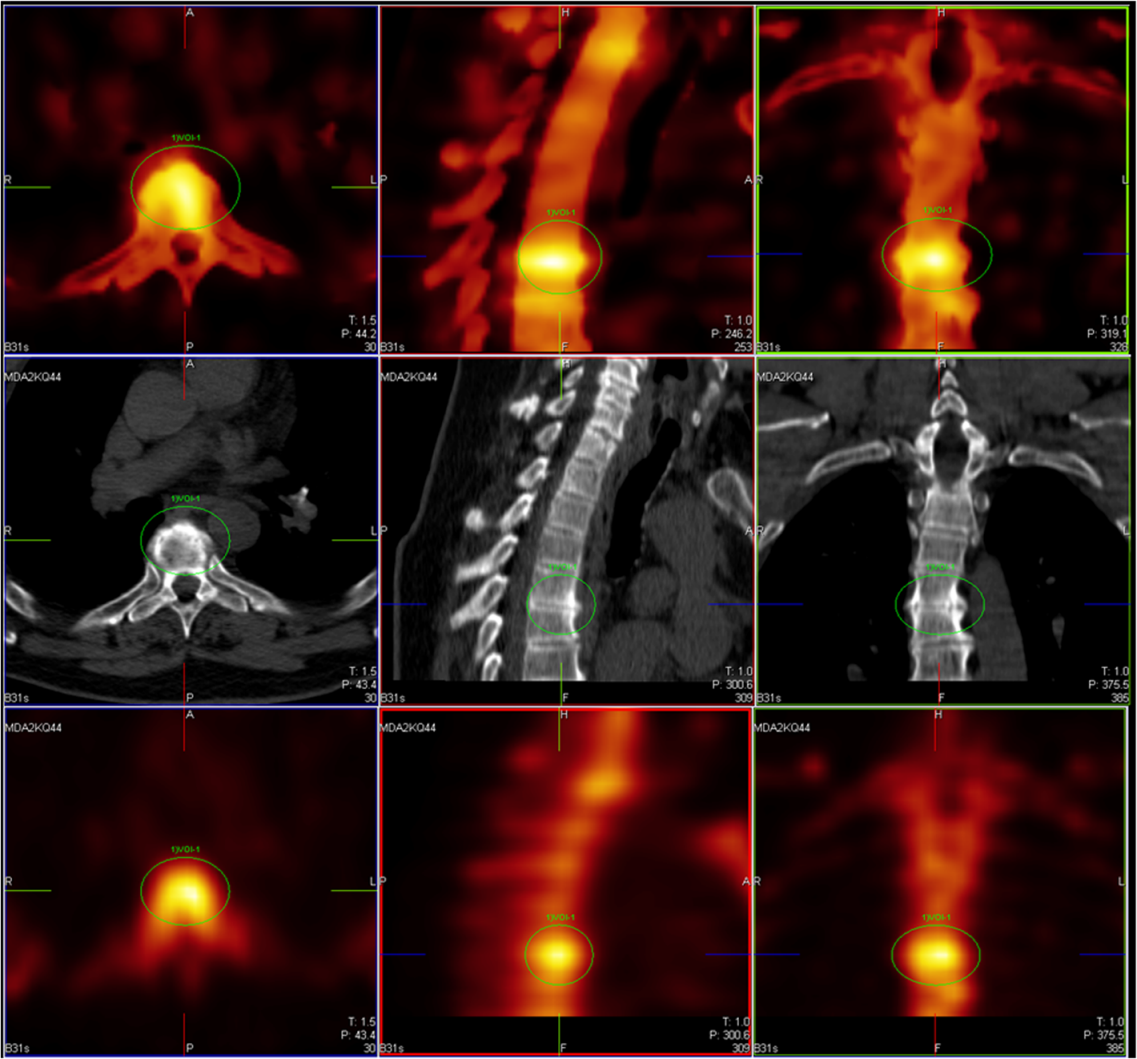
Reduction of false positive findings with xB. Despite some suboptimal xB images, five of nine F3D reads were deemed malignant and thus as false positive relative to the surrogate ground truth. In this case, only two of nine reads were deemed as malignant using xB, yet all readers concurred that the lesion was benign using xB/CT. [MDAKQ44 L1]

**Fig. 6:**
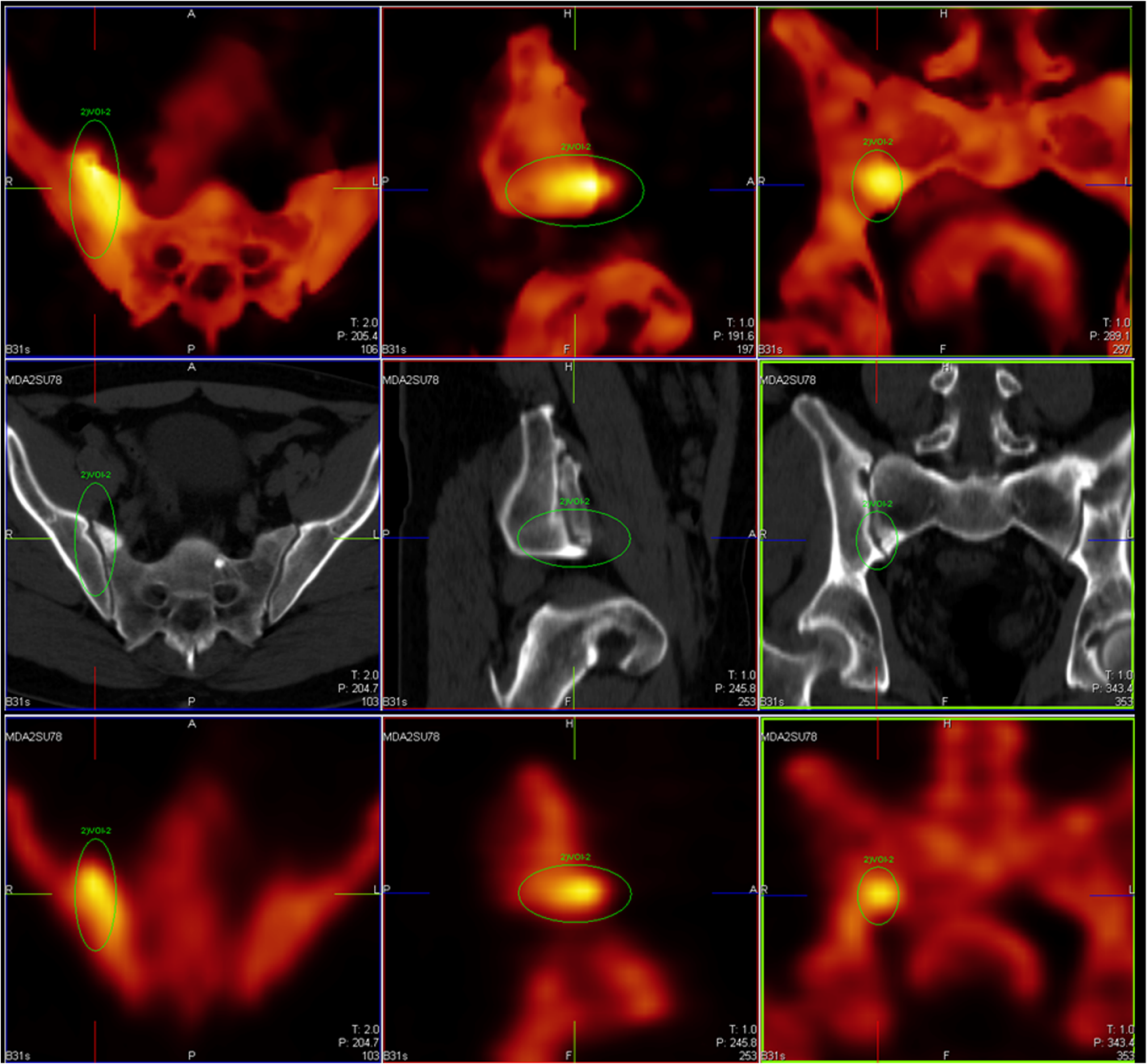
Signature of small spatio-temporal inconsistency (revealed by the “halo” of activity arising in soft tissue adjacent to the bone). This shows a limitation of the method as implemented in this version, i.e., without motion correction of emission image. [MDASU78 L2]

**Fig. 7:**
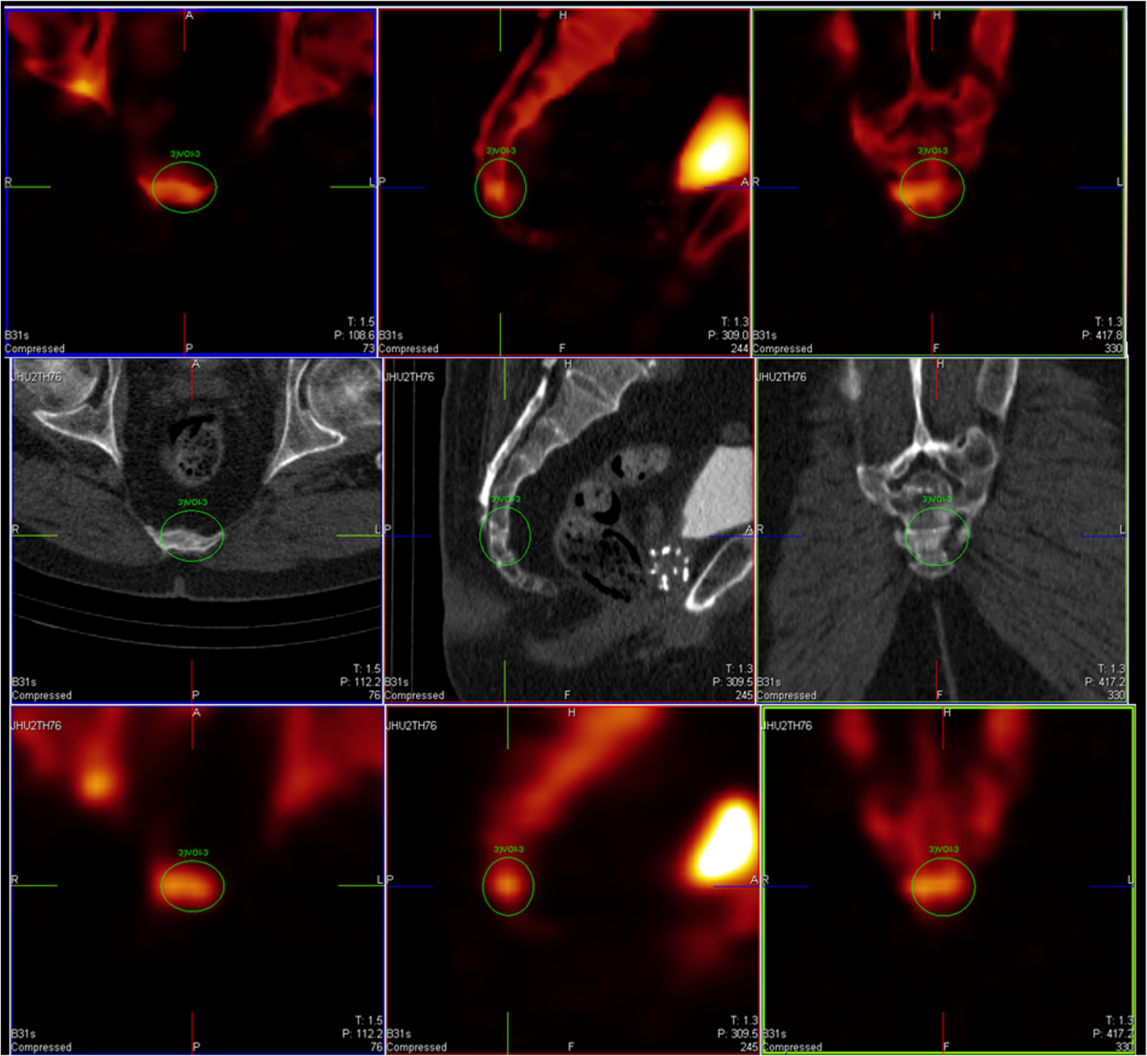
Findings in very small structures for (upper row) xB versus F3D (bottom row). These images show the signature of small spatio-temporal inconsistency and uncorrected partial volume effects in the present version. [JHUTH76 L3]

In the following, we present a few illustrative examples to clarify the rather dry statistical analysis presented above (Figs. 3, 4, 5, 6, and 7). We always depict xB in the first row, CT in the middle row, and F3D at the bottom. Figures 3, 4, 5, and 6 showcase the ability of our method to improve confidence in the reader. Figure 3 shows a clearly visible lesion unilateral to the ileosacral joint, with its center below the joint surface. This is much clearer in xB than in F3D, constituting a case with change of diagnosis from benign to malign, read as benign by only 2/9 readers in F3D, but by all readers as malign in xB. Figure 4 shows a clearly visible lesion, read as benign by two readers in F3D, and as malign by all readers in xB, with confirmation by CT. Figure 5 shows a clearly visible lesion, read as malign by 5/9 readers in F3D, but by only two readers in xB, one in F3D/CT, and none in xB/CT, with final diagnosis as benign. Figure 6 shows a clearly visible lesion, for which xB and F3D were inconclusive, and with increased bone density (osteoplastic metastasis). Here, the addition of CT information resolved the difficulty due to motion during the scan, as depicted but the telltale signature of “halo”—activity (especially for small lesions in xB). This motion artifact seemingly arises in soft tissue adjacent to bone, albeit at much lower concentration, whereas the movement is not captured in the CT. Figure 7 shows a clearly visible lesion, read as malignant by one reader in F3D, but by four in xB. In this case (judged as benign), the poor definition of bone structure by CT is due to very low CT dose as well as low uptake contrast.

### Quantitative assessment

**Fig. 8:**
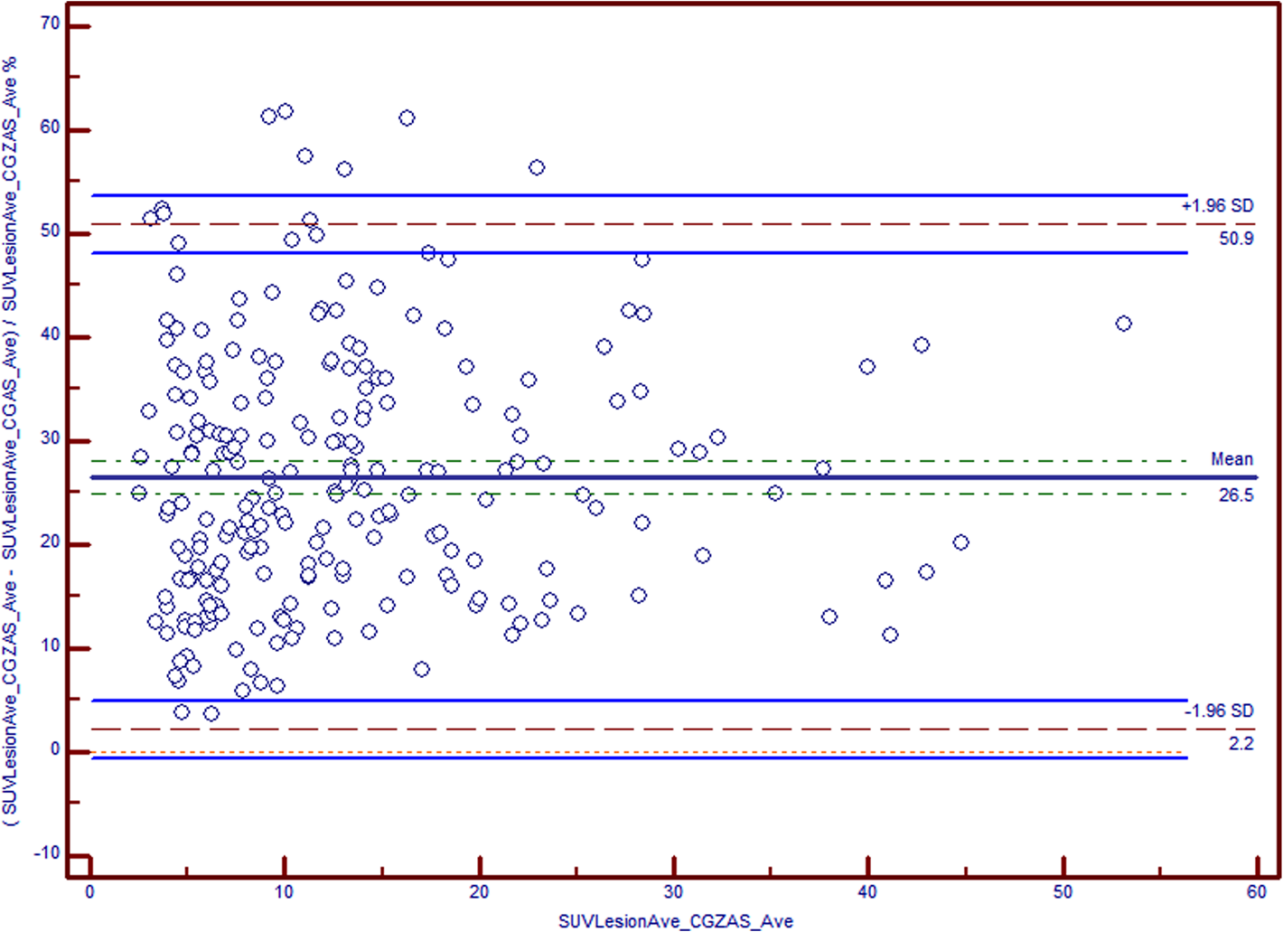
Bland-Altmann plot comparing SUV from zonal and non-zonal reconstructions

An analysis of the average uptake of MDP or DPD in the different tissue types as segmented by zones shows that the method captures the expected uptake ratio. For instance, the ratio of the means of the uptake in zone 5 (cortical bone) to zone 3 (soft tissue) is about 13:1 (Table 8). The mean CGZAS SUV is about 26.5% greater than the mean CGAS SUV, as shown in Fig. 3. In particular, small lesions show higher uptake to CGZAS, which is consistent with phantom measurements; the difference by method is explicable as an inherent property of the zonal method, whereby all voxels of the same zone are normalized as an ensemble (Fig. 8). This has the effect of making a partial volume correction (PVC), without being explicitly defined as such (Vija 2017). In Table 9 (row 1: none), the ROC analysis shows that the AUC for CGZAS (85.4%) numerically exceeds that of CGAS (80.9%), a difference that is statistically significant (Table 10). The difference in AUC also shows that one and ten realizations did not yield significantly different AUCs or ROCs, indicating a low level of statistical noise, as expected from the pilot trial. The SUV criterion is > 11.6 for CGZAS versus only > 9.4 for CGAS, indicating a significantly higher separation criterion for CGZAS between benign and malignant lesion than previously reported (Ma et al. 2013). Pure SUV-based classification may not always be appropriate, as the location of the lesion influences physiological uptake. Thus, using a simple location-based classification of “joint” or “not joint” in addition to SUV increased the AUC only when “not joint” SUVs are selected. For the case of CGZAS, the “not joint” AUC increased to 90.3%, thus approaching the visual interpretation, with a criterion of > 9.9. In the case of CGAS, AUC increased to 86.4%, also approaching the visual interpretation, and with a criterion of > 8.6 (Table 9: row 2). These differences between the ROC and respective AUC from CGZAS and CGAS are statistically significant (*p* < 0.05; Table 10). When classification is done at joints, we obtained for the threshold criterion of > 13.4 with an AUC of 83.2% for CGZAS, versus a criterion of > 9.4 and 80.0% AUC for CGAS. The differences are statistically significant (*p* < 0.05; Table 9: row 3, Table 10). When further classifying the data using also lesion volume and selecting constraining conditions (volumes ≤ 6 ml and not joint), the comparison of ROC curves shows a nearly significant increase of AUC to 81.2% for CGAS and 87.7% for CGZAS (*p* = 0.06 when the lesion volume is defined by CGAS only). The difference disappeared when the lesion volume is defined by CGZAS, as expected given the higher resolution image. However, when only considering test volumes ≤ 6 ml, the AUC is 94% for both ROC curves (*P* = 0.93; Table 9: rows 4–6, Table 10). This may explain why the visual read gives greater concordance, confidence, and accuracy. Namely, the benefit of a visual read stems from a better depiction of smaller lesions, thus improving their interpretability. In addition, it is noteworthy that a simple classification and quantitative analysis with an SUV threshold performs about as well as do experienced readers (Table 5 and 10).

**Table 8:** Absolute quantitative uptake in tissue zones

	Zone 1—Lung and lower density	Zone 2—Adipose	Zone 3—Soft tissue	Zone 4—Spongy bone	Zone 5—Cortical bone
Mean (kBq/ml)	0.62	1.32	3.52	27.5	44.3
95% CI	0.52–0.72	1.15–1.49	3.09–3.96	24.9–30.0	38.8–49.8
SD	0.44	0.73	1.92	11.3	24.1

**Table 9:** Summary of SUV-based sensitivity and specificity

	Constraints	Sample size	Positive group	Modality^a^	AUC	SE^b^	95% CI^c^	z-statistic	Significance level *P*	Youden index *J*	95% Cl^d^	Associated criterion (SUV)	95% CI^d^
1)	None	228	82	xB	0.854	0.026	0.801–0.897	13.69	< 0.0001	0.538	0.423–0.597	> 11.63	8.6–17.1
				xQ	0.809	0.031	0.752–0.858	9.96	< 0.0001	0.529	0.399–0.627	> 9.4	8.54–12.32
2)	Not joints	120	63	xB	0.903	0.028	0.835–0.949	14.27	< 0.0001	0.698	0.555–0.780	> 9.86	8–11.63
				xQ	0.864	0.034	0.780–0.919	10.68	< 0.0001	0.658	0.530–0.771	> 8.61	4.41–9.05
3)	Joints	108	19	xB	0.832	0.056	0.748–0.897	5.95	< 0.0001	0.640	0.437–0.790	> 13.42	9.25–13.87
				xQ	0.800	0.056	0.712–0.870	5.34	< 0.0001	0.584	0.373–0.734	> 9.4	9.18–9.47
4)	Not joints, CGAS volume ≤ 6 ml	45	29	xQ	0.812	0.063	0.668–0.913	5.00	< 0.0001	0.552	0.296–0.662	> 8.2	5.25–8.2
				xB	0.877	0.053	0.745–0.956	7.16	< 0.0001	0.6467	0.400–0.806	> 9.11	6.90–11.29
5)	Not joints, CGZAS volume ≤ 6 ml	64	34	xQ	0.830	0.051	0.716–0.913	6.54	< 0.0001	0.529	0.294–0.624	> 8.41	5.14–8.41
				xB	0.868	0.045	0.760–0.939	8.13	< 0.0001	0.653	0.4290–0.8000	> 9.11	7.66–11.29
6)	Not joints, CGZAS volume ≥ 6 ml	56	29	xQ	0.941	0.034	0.843–0.986	12.85	< 0.0001	0.854	0.706–0.963	> 9.28	6.74–9.28
				xB	0.940	0.034	0.842–0.986	13.00	< 0.0001	0.852	0.633–0.926	> 9.86	8–11.63

^a^Ten realizations

^b^DeLong (DeLong et al. 1988)

^c^Binomial exact

^d^BC a bootstrap interval (1000 iterations)

**Table 10:** Comparison of ROC’s: area under the curve (AUC)

	Constraints	Sample size	Positive group	Negative group	SUV lesion average of reconstruction modality	AUC	SE^a^	95% CI^b^
1)	None	228	82	146	CGAS_Ave	0.809	0.0311	0.752–0.858
					CGZAS	0.855	0.0256	0.803–0.898
					CGZAS_Ave	0.854	0.0258	0.801–0.897
2)	Joints	108	19	89	CGAS_Ave	0.8	0.0561	0.712–0.870
					CGZAS_Ave	0.832	0.0558	0.748–0.897
3)	Not joints, CGAS Volume ≤ 6 ml	45	29	16	CGAS_Ave	0.812	0.0625	0.668–0.913
					CGZAS_Ave	0.877	0.0527	0.745–0.956
4)	Not Joints, CGZAS volume ≤ 6 ml	64	34	30	CGAS_Ave	0.83	0.0505	0.716–0.913
					CGZAS_Ave	0.868	0.0452	0.760–0.939
5)	Not Joints, CGZAS volume ≥ 6 ml	56	29	27	CGAS_Ave	0.941	0.0343	0.843–0.986
					CGZAS_Ave	0.94	0.0338	0.842–0.986
Pairwise comparison of ROC’s: SUV-based lesion assessment								
	Constraints	SUV lesion average of reconstruction modality	Difference between areas	SE^a^	95% CI^b^	z-statistic	Significance level	
1)	None	CGAS_Ave ~ CGZAS	0.046	0.015	0.016–0.076	2.97	*p* = 0.0030	
		CGAS_Ave ~ CGZAS_Ave	0.045	0.013	0.019–0.070	3.394	*p* = 0.0007	
		CGZAS ~ CGZAS_Ave	0.0012	0.009	− 0.016-0.018	0.137	*p* = 0.8912	
2)	Joints	CGAS_Ave ~ CGZAS_Ave	0.033	0.017	0.0003–0.07	1.977	*p* = 0.0480	
3)	Not joints, CGAS volume ≤ 6 ml	CGAS_Ave ~ CGZAS_Ave	0.065	0.034	− 0.003-0.132	1.881	*p* = 0.0600	
4)	Not joints, CGZAS volume ≤ 6 ml	CGAS_Ave ~ CGZAS_Ave	0.037	0.026	− 0.014-0.09	1.431	*p* = 0.1525	
5)	Not joints, CGZAS volume ≥ 6 ml	CGAS_Ave ~ CGZAS_Ave	0.0006	0.007	− 0.014-0.015	0.0861	*p* = 0.9314	

^a^DeLong (1988)

^b^Binomial exact

## Discussion

Results of this study show that the quantitative reconstruction method xSPECT Bone, which uses CT information to delineate tissue boundaries and assign voxels to five tissue classes or zones, can be beneficial for the clinical reader. In the first part of our study, we made an assessment based purely on visual interpretation. We first showed that readers are more consistent and more confident in the xSPECT Bone read as compared to the current clinical state-of-the art method, and we then showed that the reads were more correct with xSPECT Bone relative to a consensus-based “truth,” wherein all relevant medical information are available for interpretation.

Lack of trustworthy standard of proof plagues studies aiming to assess diagnostic performances of ^99m^Tc-disphosphonate SPECT/CT. However, in cooperation with experienced interventional radiologists, bone biopsies are now performable in patients, with a high cost-effective yield. Unfortunately, biopsy proved impossible in this multi-center trial due to difficulties in agreeing upon to protocol standardization, and the matter of patient consent. Compiling published studies of bone SPECT/CT reveal that the fewer biopsies in the study, the high specificity that is reported (MAd and Paycha 2015). As this confirmatory invasive procedure could not be performed in our study, we rely upon the surrogate gold standard (Naaktgeboren et al. 2013).

We saw the largest benefit of xSPECT Bone when only the nuclear images were shown, and most particularly when only small lesional volumes-of-interest (≤ 6 ml) were involved. In these cases, xSPECT Bone significantly outperformed Flash3D. Upon adding CT to the interpretation, the relative benefit of the new method remained, but was of lesser magnitude.

In the second part of the study, we used a quantitative ROC analysis to define SUV threshold values, which can further assist the visual read, or may indeed prove to serve in a stand-alone role once more supporting data is collected. For the present, we found that SUV threshold values were in the range of 9–13, depending on the various constraints imposed on the ROC analysis. These thresholds are consistent with previous findings, although there remains a need for further validation before their translation to clinical practice.

In designing the study, we attempted to control for relevant variables, although there remains scope for certain improvements in the reconstruction tool and the data analysis pipeline. The primary point of potential contention in this study lies in establishing ground truth based on consensus readings, a matter which has been discussed in the literature (Bankier et al. 2010). Indeed, as noted above, the only definitive approach entails either lesion biopsy or longitudinal monitoring through repeat scanning. Both approaches are logistically very difficult; in the present context, the consensus reading ultimately defaulted to the CT image interpretation as the tiebreaker. This implies that none of the methods herein can surpass the accuracy of the SPECT/CT read, thus explaining why fusion of the two reconstruction methods with CT showed little difference in AUC, whereas there was a significant difference when CT was omitted from consideration. Thus, defining ground truth by some more definitive means such as biopsy would naturally help to overcome ceiling or circularity effects inherent in the present design. A second critical point is the lack of explicit PVC, which likely introduces a systematic underestimating bias when establishing the SUV threshold for small lesions. Approaches to PVC typically assume a uniform tracer uptake within the structure and require knowledge of the exact lesion or structure boundary. The latter issue is partially addressed through our approach, although there remains uncertainty about the heterogeneity of bone uptake itself, as perhaps evidenced by the evident physiological difference in joint and non-joint signals. While we shall address this issue in future experiments, we do not believe it to be a major limitation in the present context. Furthermore, the classification threshold of 6 ml was determined by trial and error; a more elaborate analysis would be needed to sample through the entire FOV for better-validated volume thresholds. This is a very complex topic, in part due to the size-dependence of lesion avidity and heterogeneity. We addressed the statistical uncertainty of our method using a bootstrap approach, albeit with only ten noise realizations. Our study included only subjects with little or no visible motion, based on lack of any conspicuous mis-registration artifacts arising from motion. In the absence of an automated motion correction and registration method within the image reconstruction (Vija and Cachovan 2017), the best approach for now entails coaching patients to reduce body motion during scan.

Despite these shortcomings, we find compelling strength in this study, due not only to the large number of patients and the lesion population but also due to the large number of independent and skilled readers from five clinical departments, all making informed and expert judgments of image quality and diagnostic benefit. The merits of the study are robust to exclusion of the extra-modal information, as the quantitative aspect alone reveals a useful SUV threshold range for diphosphonate SPECT bone imaging. Finally, after instruction and training with only a few patients (5–10), all readers were able read the images, grew accustomed to the novel noise structure and unfamiliar multi-modal resolution of the images, and could consistently identify specific artifacts, such as those due to motion or presence of non-biological material.

## Conclusion

The incorporation of CT-based high-resolution tissue zones in the reconstruction of SPECT bone imaging imparted improved image quality and higher reader concordance as compared to conventional SPECT with CT attenuation, scatter compensation and distance-dependent resolution recovery. xSPECT Bone provides better resolution and detects smaller lesions (6 ml) and the CT component helps in better characterization. After viewing a few training cases, all readers adapted to the unfamiliar presentation of the hybrid resolution images, the noise texture, and lesion acuity, while learning to identify a new type of motion artifacts and adjust their reading appropriately. The capacity for absolute quantification yielded critical threshold numbers for SUV is a remarkable aspect of our approach, as is the improved differentiation and localization of lesions, which resulted in AUC values comparable to those in an expert visual reading. However, SUV is not currently usable as an independent marker in classifying benign and malignant lesions and further evaluation with follow-up scans or biopsy gold standard might enable its use in routine clinical practice. Further investigations of the impacts of motion, mis-registration and PVC are in progress. The improvements obtained with the xSPECT Bone method reside in the capacity to better localize the foci, and the superior visualization and quantification of lesions with effective volumes less than about 6 ml.

## Abbreviations

2D: Two-dimensional
3D: Three-dimensional
^57^Co: Cobalt-57
99 m Tc: (Metastable) technecium-99
ARC: Advanced research computer
AUC: Area under the curve
BMI: Body mass index
Bq/ml: Radioactivity concentration or Becquerels (decays per second) per milliliter
CGAS: Conjugate gradient, attenuation and scatter
CGZAS: Conjugate gradient, t zonal, attenuation and scatter
CL: Confidence level
CT: Computer tomography
DPD: 99mTc-dicarboxypropane diphosphonate
EMI: Extra modal information
F3D: Flash 3D
FOV: Field-of-view
GUI: Graphical user interface
IMI: Intra modal information
IRB: Investigational review board
keV: Kilo eletctronvolt
mA: Milli-ampere
MBq: Megabecquerel
MDP: 99mTc-methylene diphosphonate
MIP: Maximum-intensity-projection
MTF: Modulation transfer function
NIST: National Institute of Standards and Technology
PVC: Partial volume correction
ROC: Receiver operator characteristics
s: Seconds
SPECT: Single-photon emission computed tomography
SUV: Standard uptake value
SUVc: The criterion standard uptake value
VOI: Volume of interest
xB: xSPECT Bone
xQ: xSPECT Quant

## Footnotes

1 A detailed description of this definition is beyond the scope of this paper.
